# Characteristics and Expression Pattern of *MYC* Genes in *Triticum aestivum*, *Oryza sativa*, and *Brachypodium distachyon*

**DOI:** 10.3390/plants8080274

**Published:** 2019-08-08

**Authors:** Shoukun Chen, Hongyan Zhao, Tengli Luo, Yue Liu, Xiaojun Nie, Haifeng Li

**Affiliations:** State Key Laboratory of Crop Stress Biology for Arid Areas, College of Agronomy, Northwest A&F University, Yangling 712000, China

**Keywords:** wheat, rice, *Brachypodium distachyon*, MYC, bHLH

## Abstract

Myelocytomatosis oncogenes (MYC) transcription factors (TFs) belong to basic helix-loop-helix (bHLH) TF family and have a special bHLH_MYC_N domain in the N-terminal region. Presently, there is no detailed and systematic analysis of MYC TFs in wheat, rice, and *Brachypodium distachyon*. In this study, 26 TaMYC, 7 OsMYC, and 7 BdMYC TFs were identified and their features were characterized. Firstly, they contain a JAZ interaction domain (JID) and a putative transcriptional activation domain (TAD) in the bHLH_MYC_N region and a BhlH region in the C-terminal region. In some cases, the bHLH region is followed by a leucine zipper region; secondly, they display tissue-specific expression patterns: wheat *MYC* genes are mainly expressed in leaves, rice *MYC* genes are highly expressed in stems, and *B. distachyon MYC* genes are mainly expressed in inflorescences. In addition, three types of *cis*-elements, including plant development/growth-related, hormone-related, and abiotic stresses-related were identified in different *MYC* gene promoters. In combination with the previous studies, these results indicate that MYC TFs mainly function in growth and development, as well as in response to stresses. This study laid a foundation for the further functional elucidation of *MYC* genes.

## 1. Introduction

bHLH (basic helix-loop-helix) family is the second largest plant transcription factor (TF) family. They are characterized by a bHLH domain, which consists of 50–60 amino acids that form two distinctive regions, the basic region and HLH (helix-loop-helix) region [1]. The basic region functions as a DNA-binding motif, while the HLH region forms two amphipathic α helices with a linking loop [1,2,3]. In the bHLH family, there are some special members, named myelocytomatosis oncogenes (MYC). These members are characterized by a so-called bHLH_MYC_N region in the N-terminal and a bHLH region in the C-terminal [1,2,4,5].

*MYC* genes function in various physiological and molecular processes, especially in growth and development. In *Arabidopsis thaliana*, *MYC2* functions synergistically with *MYC3* and *MYC4* in regulating leaf senescence [6], root elongation [7], stamen development [8], seed production, and seed storage protein accumulation [8,9], as well as chlorophyll degradation [10]; other *Arabidopsis MYC* genes, like *ALCATRAZ* functions in cell separation in fruit dehiscence [11], *SPATULA* controls development of carpel margin tissues [12], and *ABORTED MICROSPORES* (*AMS*) plays a crucial role in tapetum cell development and pollen wall formation [13]. In rice (*Oryza sativa*), the orthologous *AMS* gene *Tapetum Degeneration Retardation* (*TDR*) is necessary for tapetum degradation and anther development [14]; *OsMYC2* is expressed in all tissues, and highly expressed in the spikelets and floral organs [15]. It regulates spikelet development through the interaction with OsJAZ1 and the activation of the downstream gene *OsMADS1* [16].

*MYC* genes are also involved in plant secondary metabolism. For example, overexpression of *Arabidopsis MYC3* and *MYC4* result in more anthocyanin accumulation [17], and *MYC2*, *MYC3*, and *MYC4* regulate glucosinolate biosynthesis [18]. Wheat (*Triticum aestivum*) *MYC1* and barley (*Hordeum vulgare*) *Myc1* (*HvAnt2*) regulate anthocyanin synthesis in pericarp too [19,20]. Additionally, *NbMYC2* regulates alkaloid biosynthesis [21], and *SmMYC2* regulates the phenolic acid biosynthesis in *Salvia miltiorrhiza* [22], while *TcJAMYC1/2/4* negatively regulate the expression of genes associating with paclitaxel biosynthesis in *Taxus cuspidate* [23].

Meanwhile, *MYC* genes play important roles in response to abiotic and biotic stresses. For example, the expression of *AtMYC2* is induced by drought and salt stresses [24], and overexpressing this gene improves osmotic stress tolerance [25]. *Arabidopsis* MYC67 and MYC70 interact with ICE1 and negatively regulate cold tolerance in *Arabidopsis* [26]. Additionally, rice *OsMYC2* negatively regulates JA-mediated resistance to a necrotrophic pathogen [27,28].

Especially, several *MYC* genes were reported to associate with JA (jasmonate) signal. *Arabidopsis MYC2*, *MYC3*, *MYC4*, and *MYC5* are master regulators in JA signal pathway [17,27,29,30]. *Arabidopsis JASMONATE-INSENSITIVE1* is essential for jasmonate-regulated defense responses [27]. In addition to regulating spikelet development via the JA signal [16], *OsMYC2* mediates numerous defense-related transcriptional changes via JA signaling [31].

Wheat (*T. aestivum*) and rice (*O. sativa*) are important cereal crops, and *B. distachyon* is a grass model plant and has a close genetic relationship with wheat [32]. In this study, *TaMYC*, *OsMYC*, and *BdMYC* genes were characterized at the genome-wide level, and the expression patterns were analyzed. Meanwhile, the specific domain of MYC proteins and the *cis*-elements in the promoters were identified. Based on these results, we summarized the characteristics of MYC TFs. This study laid a foundation for further functional elucidation of *MYC* genes. 

## 2. Results

### 2.1. Wheat, Rice, and B. distachyon MYC TFs

**Figure 1:**
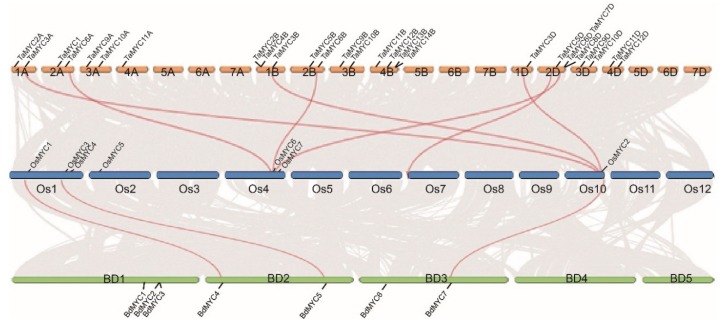
Chromosome location, homologous gene pairs of *TaMYC*, *OsMYC*, and *BdMYC* genes. Gray lines in the background indicate the collinear blocks within wheat, rice, and *B. distachyon* genomes, while the red lines highlight the homologous gene pairs.

Totally, 26 putative *TaMYC*, 7 *OsMYC*, and 7 *BdMYC* genes were identified in the relevant genomes, respectively (Figure 1), including two reported genes: *TaMYC1* and *OsMYC2* [15,19]. Among these 40 genes, 34 genes were verified by ESTs (Expressed Sequence Tags) deposited in the NCBI database, and 18 *TaMYC* genes constitute 6 sets, every set includes three homologous genes in A, B, and D subgenomes, respectively.

Except for *TaMYC1* and *OsMYC2,* other 25 *TaMYC* genes were named as *TaMYC2A* to *TaMYC12B* according to their distribution on chromosomes and genomic homology; other 6 *OsMYC* and 7 *BdMYC* genes were named as *OsMYC1*, *OsMYC3* to *OsMYC7,* and *BdMYC1* to *BdMYC7* based on their chromosomal localization (Figure 1).

The physical features of these MYC TFs were predicted. In wheat, the protein length varies from 456 (TaMYC7A) to 699 (TaMYC4D) amino acids; the PI (Isoelectric Point) varies from 4.96 (TaMYC6D) to 8.73 (TaMYC9D); and the molecular weight varies from 49.17 kDa (TaMYC9A) to 63.69 kDa (TaMYC7D). In rice, the protein length varies from 473 (OsMYC7) to 904 (OsMYC4) amino acids; the PI varies from 4.68 (OsMYC5) to 8.36 (OsMYC3); and the molecular weight varies from 50.67 kDa (OsMYC3) to 97.39 kDa (OsMYC4). In *B. distachyon*, the protein length varies from 470 (BdMYC5) to 706 (BdMYC7); the PI varies from 4.53 (BdMYC6) to 7.22 (BdMYC5); and the molecular weight varies from 50.43 kDa (BdMYC5) to 76.24 kDa (BdMYC7). The protein subcellular localization was predicted by CELLO web server [33] and results showed that 26 MYC proteins locate in the nucleus, while other 14 MYC proteins locate in the chloroplast, cytoplasm, mitochondria. The detailed information is listed in Table S1.

### 2.2. Sequence Alignment and Phylogenetic Tree of MYC TFs

**Figure 2:**
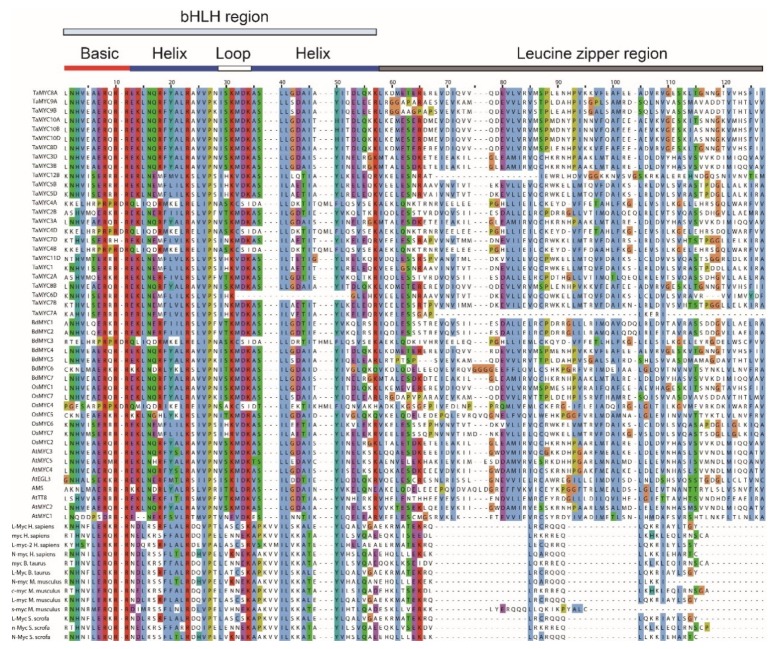
Alignment of the 61 different myelocytomatosis oncogenes (MYC) proteins from plants and animals. Including 40 MYCs in this study, 8 *Arabidopsis* MYCs, and 13 animal MYCs proteins from previous studies as described above.

Based on previous studies [13,18,34,35,36,37,38], protein sequences of 13 known animal MYCs and 8 known *Arabidopsis* MYCs obtained from GenBank [39], and sequences of 40 MYCs in this study were used to perform sequence alignment. Multiple sequence alignment identified a distinct N-terminal bHLH_MYC_N region (Figure S1). As shown in Table S2, the consensus ratio of 56 conserved amino acid residues is more than 50% in the bHLH_MYC_N domains. Leu-100, Trp-113, Tyr-115, Trp-119, and Leu-147 are identical in these 40 MYC TFs, indicating that these amino acids are key component of bHLH_MYC_N domain. More importantly, one JAZ interaction domain (JID) and one putative transcriptional activation domain (TAD) were identified in the bHLH_MYC_N domain. Furthermore, a bHLH domain and a leucine zipper region were found in the C-terminal region (Figure 2 and Figure S1).

In animals, MYC proteins are key regulators of mammalian cell proliferation that activate genes as part of a heterodimeric complex with the protein Max [40]. As shown in Figure S2, the alignment of 6 known Max proteins (Genbank number: NP_660092.1 [*Homo sapiens*], XP_002613458.1 [*Branchiostoma floridae*], XP_008195890.1 [*Tribolium castaneum*], NP_510223.1 [*Caenorhabditis elegans*], NP_001099103.1 [*Bos taurus*], NP_002373.3 [*Homo sapiens*]) and other MYC proteins showed that they are highly conserved in bHLH domains, while poorly conserved in leucine zipper region. 

According to the alignment of bHLH domains in rice and *Arabidopsis* bHLH proteins [41], some amino acids residues, such as Val-960 and Ala-962 in the basic region, Asn-972 and Val-981 in the first Helix, Ser-986, Lys-987, Met-1006, and Asp-1008 in the Loop, and Asp-1017 in the second Helix, are only found in plant MYC proteins (Figure 2 and Table S3). In addition, although the bHLH-ZIP domain is commonly present in some human c-Myc and Max proteins [42], such as myc_*H. sapiens*, myc_*B. taurus*, c-myc_*M. musculus*, n-Myc_*S. scrofa* in Figure 2, they were lowly conserved in the leucine zipper region. 

**Figure 3:**
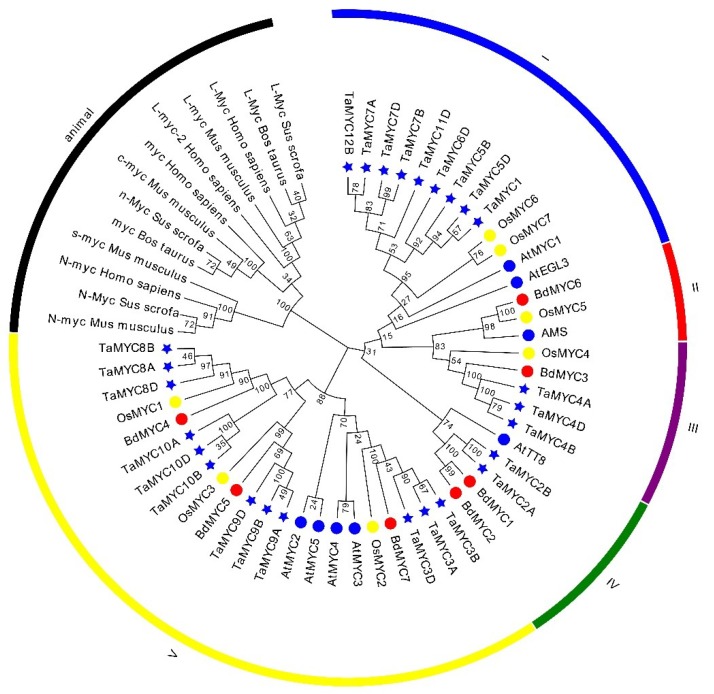
Phylogenetic tree of 61 MYC transcription factors (TFs). There are 48 plant MYCs and 13 known animal MYCs. The Ta, Os, Bd, and At represent wheat, rice, *B. distachyon,* and *Arabidopsis,* respectively.

To understand the evolutionary relationships of MYC TFs, a neighbor-joining (NJ) phylogenetic tree was constructed based on the full-length alignment of 40 putative MYCs, 13 known animal MYCs, and 8 known *Arabidopsis* MYC. As shown in Figure 3, these MYC TFs are classified into six sub-groups with highly bootstrap values. The class V is the largest while the class II was the smallest. There are 9 TaMYCs, 2 OsMYCs, and 2 AtMYCs in class I; 1 OsMYC, 1 BdMYC, and 1 AtMYC in class II; 3 TaMYCs, 1 OsMYC, and 1 BdMYC in class III; 2 TaMYCs, 2 BdMYCs, 1 AtMYC in class IV; 12 TaMYCs, 3 OsMYCs, 3 BdMYCs, and 4 AtMYCs in class V; and 13 known animal MYCs in class “animal”.

### 2.3. Gene Structures and Conserved Motifs

**Figure 4:**
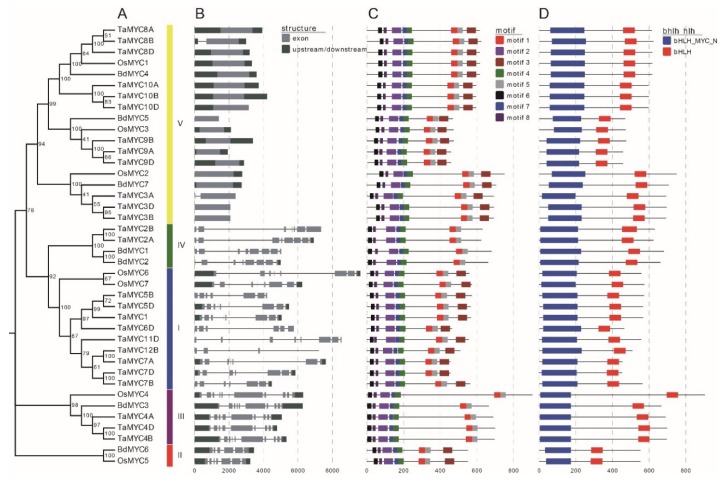
(**A**) Phylogenetic relationships, (**B**) gene structures, (**C**) motifs, and (**D**) conserved regions of wheat, rice, and *B. distachyon* MYCs. The tree was constructed with 1000 bootstrap replications using MEGA7 based on the full-length protein sequence. The exon–intron structure of these genes was graphically displayed by the Gene Structure Display Server using the coding sequences (CDS) and DNA sequences of *MYC* genes. The protein sequence of MYC proteins was used to predict the conserved motifs/region by using the MEME Suite web server.

We used genomic DNA sequences and CDS (coding sequences) to analyze the gene structure (Figure 4B). Gene structures of *TaMYC*, *OsMYC*, and *BdMYC* genes are similar within the same subgroup. In wheat, the exon number ranges from 1 to 11. In rice and *B. distachyon*, the exon number ranges from 1 to 10. Notably, most class V members only have one exon.

Conserved motifs are helpful to understand the functions of MYC TFs. In this study, 8 conserved motifs were identified (Figure 4C). Motifs 2, 4, 6, 7, and 8 constitute the bHLH_MYC_N domain (Figure 4D). Among these five motifs, motifs 6 and 8 form JID and motif 2 composes TAD. JID consists of approximately 90 amino acids, including a specific motif (W-[TN]-Y-[AG]-[IVL]-[FYL]-W-X(6,19)-L-[GT]-W-[GK]-[DE]-G). TAD includes approximately 70 amino acids and a specific motif ([VL]-[TG]-[DEG]-[TA]-E-[WML]-[FY]-[FY]-X(2)-[SC]-[MA]-X(3)-F-X(4)-G-[LAG]-P-G-X(9)-W). 

In addition, as MYCs belong to bHLH TF families [3,41,43], motifs 1 and 5 constitute bHLH domain (Figure 4D), and motif 3 constitutes the leucine zipper in class I, II, and V members. 

### 2.4. Synteny and Homologous Gene Pairs

Gene duplication events include tandem duplication and segmental duplication. We analyzed the gene duplication by using the MCScanX software [44]. As shown in Table S4, only one segmental duplication pair (TaMYC6D-TaMYC7D) was found.

In addition, orthologous between *MYC* genes in wheat, *B. distachyon*, *A. thaliana*, rice, *H. vulgare*, *Sorghum bicolor*, and *Zea mays* were also investigated (Table S5). A total of 12, 0, 19, 7, 22, and 28 orthologs and orthologous gene pairs between wheat and *B. distachyon*, *A. thaliana*, rice, *H. vulgare*, *S. bicolor*, and *Z. mays* (Table S5); 2, 5, 4, 6, and 7 orthologs and orthologous gene pairs between *B. distachyon* and *A. thaliana*, rice, *H. vulgare*, *S. bicolor*, and *Z. mays*; 6, 3, 3, and 2 orthologs and orthologous gene pairs between rice and *A. thaliana*, *S. bicolor*, *H. vulgare*, and *Z. mays.* These results suggested that *MYC* genes of monocots have strong relationships.

### 2.5. Identified Cis-Elements in MYC Gene Promoters

**Figure 5:**
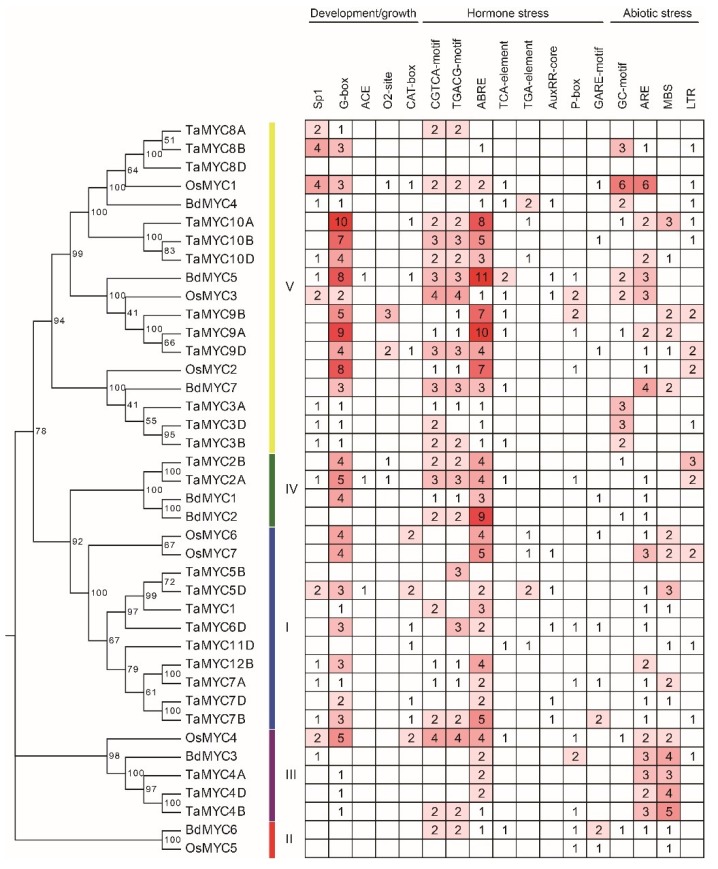
Main *cis*-elements in *TaMYC*, *OsMYC*, and *BdMYC* gene promoters.

We also identified the *cis*-elements in the 2-kb promoters of *MYC* genes using the PlantCARE web tool [45]. As shown in Figure 5, three types of *cis*-elements, which are related to plant growth/development, hormone, and abiotic stresses, were identified. The *cis*-elements related to growth/development include light-responsive element G-box (CACGTC) [46] and Sp1 (GGGCGG) [47], the metabolism regulation related *cis*-element O2-site [48], and the meristem expression related *cis*-element CAT-box [49]. The *cis*-elements related to hormones include the methyl jasmonate (MeJA)-responsive elements CGTCA-motif [50] and TGACG-motif [51], the abscisic acid (ABA)-responsive element ABRE (ACGTG) [52], the gibberellin(GA)-responsive element GARE-motif [53], the auxin-responsive elements TGA-element [54] and AuxRR-core (GGTCCAT) [55], and the salicylic acid (SA)-responsive element TCA-element (CCATCTTTTT) [56]. The *cis*-elements associated with abiotic stresses include adaptive elements such as drought-inducibility element MBS (CAACTG) [57], low-temperature responsiveness element LTR (CCGAAA) [58], anaerobic induction ARE (AAACCA) [59], and anoxic specific inducibility element GC-motif (A/CGCCGCGCA) [60] were detected in a series of members. Combined with the phylogenetic tree, these results showed that the phylogenetically similar genes shared identical *cis*-elements.

### 2.6. Expression Profiles of MYC Genes

**Figure 6:**
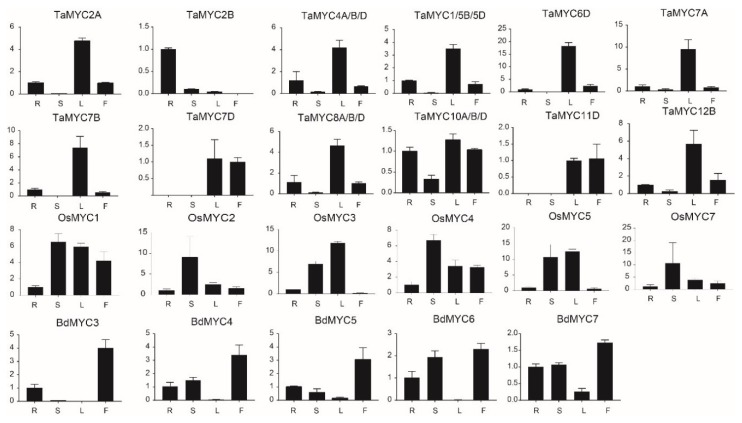
Expression patterns of *TaMYC*, *OsMYC*, and *BdMYC* genes in different tissues. R, S, L, F indicate roots, stems, leaves, and inflorescences. The horizontal and vertical coordinates stand for four different tissues and the relative expression, respectively.

We analyzed the expression patterns of *TaMYC*, *OsMYC*, and *BdMYC* genes by quantitative real-time polymerase chain reaction (qRT-PCR). Among these 40 *MYC* genes, the expression of 9 genes (*TaMYC3A/B/D*, *TaMYC9A/B/D*, *OsMYC6*, *BdMYC1*, and *BdMYC2*) was not detected in all cases. As shown in Figure 6, *MYC* genes displayed tissue-specific expression. A total of 11 *TaMYC* genes (including *TaMYC2A*, *TaMYC4A/B/D*, *TaMYC1/5B/5D*, *TaMYC6D*, *TaMYC7A*, *TaMYC7B*, *TaMYC7D*, *TaMYC8A/B/D*, *TaMYC10A/B/D*, *TaMYC11D*, and *TaMYC12B*) were highly expressed in leaves, 4 *OsMYC* genes (*OsMYC1*, *OsMYC2*, *OsMYC4*, and *OsMYC7*) were highly expressed in stems, and 5 *BdMYC* genes (*BdMYC3*, *BdMYC4*, *BdMYC5*, *BdMYC6*, and *BdMYC7*) were highly expressed in inflorescences. 

**Figure 7:**
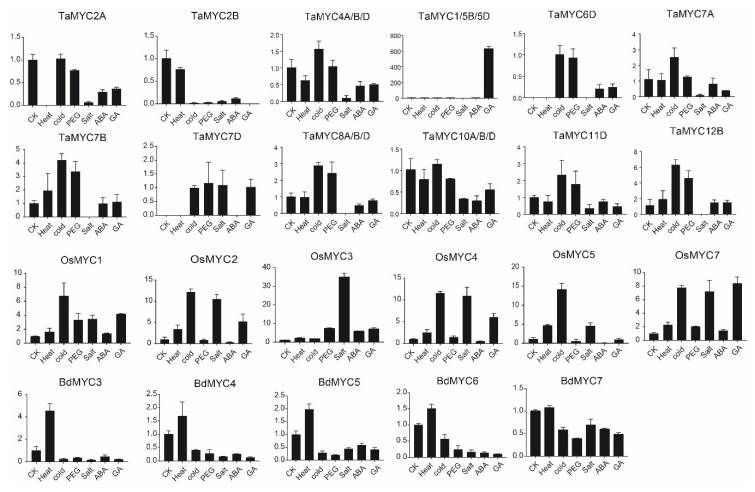
Expression patterns of *TaMYC*, *OsMYC*, and *BdMYC* genes under abiotic stresses; CK (H2O), Heat, Cold, PEG, Salt, ABA, GA indicate different treatments. The horizontal and vertical axes stand for different abiotic stresses and the relative expression, respectively.

Additionally, we also analyzed the expression of these genes in two-week seedlings with different treatments (Figure 7). Although the results showed no regularity, there are some meaningful findings. In wheat, the expression of *TaMYC1/5B/5D* is induced by GA (Gibberellin), indicating the involvement in GA signal. *TaMYC7A*, *TaMYC7B,* and *TaMYC7D* displayed different expression patterns, implying that their functions are diversified. In rice, the expression of six expressed genes is upregulated by different abiotic stresses, suggesting their important roles. In *B. distachyon*, *BdMYC3* is drastically induced by heat.

## 3. Discussion

### 3.1. The Characteristics of MYCs 

bHLH TFs are the second largest class of plant TFs [61]. Plant MYC TFs belong to the bHLH superfamily. MYC proteins have one bHLH_MYC_N domain in the N-terminal region [1]. In this domain, Leu-100, Trp-113, Tyr-115, Trp-119, and Leu-147 amino acids residues are complete conserved in all TaMYC, BdMYC, and OsMYC proteins. The bHLH_MYC_N include two domains: JID and TAD. The former is necessary for interacting with JAZ proteins, while the latter is a putative transcriptional activation domain [2]. For example, *Arabidopsis* MYC2, MYC3, and MYC4 interact with the C-terminal JAS domain of JAZ proteins through JID, and AtMYC2 recruits the mediator complex required for transcription initiation through its TAD, which specifically interacts with the activator interaction domain [62,63]. We also identified one specific motif (W-[TN]-Y-[AG]-[IVL]-[FYL]-W-X(6,19)-L-[GT]-W-[GK]-[DE]-G) in JID and one specific motif ([VL]-[TG]-[DEG]-[TA]-E-[WML]-[FY]-[FY]-X(2)-[SC]-[MA]-X(3)-F-X(4)-G-[LAG]-P-G-X(9)-W) in TAD.

The C-terminal region of *TaMYCs*, *BdMYCs*, and *OsMYCs* is conserved and includes the typical basic region and HLH domain, and most MYCs contain leucine zipper (bZIP) domain (Figure 2 and Figure S2). Previous reports showed that the basic region of MYC proteins mainly recognizes CACGTG (G-box) sequence and CATGTG sequence, both E-box DNA-binding sites (5′-CANNTG-3′), and the bHLH domain are required for the heterodimer to bind to the G-box in target genes [2]. The bHLH-ZIP domain is present in human c-Myc and Max proteins [42], which have DNA-binding activity and has been predicted to mediate protein–protein interactions. The interaction of Max and c-Myc depends on the integrity of the c-Myc HLH-Zip domain, but not the basic region or other sequences outside the domain [42]. Different from animal MYCs, plants MYCs contain a longer (about 70 amino acids) leucine zipper. Furthermore, the C-terminal leucine zipper domain can form the dimer that affects the specificity of interaction with other TFs [64]. Compared to other bHLH proteins in plants [43,65], bHLH-ZIP domain only appeared on group B which MYC and Max exist.

Compared to *bHLH* genes, *MYC* genes have three special kinds of *cis*-elements in their gene promoters. These *cis*-elements associate with their special functions. Moreover, *MYC* genes show tissue-specific expression patterns. Additionally, different from other non-*MYC-bHLH* genes which have 2 or more exons [41,43,65], most class V MYC members, which is the largest class only, have one exon.

### 3.2. Functions of MYC TFs

Previous studies showed that MYC TFs function in plant development and growth [2,9,37]. For example, *MYC* proteins function as regulators in regulating plant seed production, root elongation, leaf senescence, and stamen development [6,7,8,9]; they also regulate plant secondary metabolism and are actively involved in hormone-mediated plant growth [21,66,67]. In this study, most *MYC* genes are expressed in roots, stems, leaves, and inflorescences (Figure 6). In combination with the identification of many *cis*-elements related to plant growth and development and hormone stress, these results further suggest their functions in growth and development.

The expression profiles of many *MYC* genes indicate their probable functions in response to abiotic and biotic stresses. For example, *Arabidopsis MYC2*, *MYC3*, *MYC4*, and *MYC5* are induced by jasmonate [17,27,29,30]; MYC3 and MYC4 act additively with MYC2 to regulate defense against insect herbivory [30]; and *MYC5* regulates JA-mediated plant defenses against herbivores and is involved in JA-regulated plant resistance to pathogens [37]. Overexpression of *OsMYC2* results in bacterial blight resistance in rice [16]. In maize, the expression of two *MYC* genes, *ZmbHLH103* and *ZmbHLH104*, is significantly upregulated under drought stress [65]. In this study, the expression of 6 *OsMYC* genes is induced by different abiotic stresses (Figure 7), further indicating that *MYC* genes participate in response to abiotic stresses. Consistently, many *cis*-elements related to abiotic stresses were identified.

Taken together, we can draw a conclusion that MYC TFs mainly function in growth/development, and in response to environmental stresses.

## 4. Materials and Methods 

### 4.1. Identification of MYC TFs in Wheat, Rice, and B. distachyon

The wheat, rice, and *B. distachyon* genome sequences, protein sequences, coding sequences (CDS), and upstream 2-kb genomic DNA sequences were downloaded from Ensembl plants [68]. The chromosomal distribution of *MYC* genes was obtained from the wheat, rice, and *B. distachyon* genome annotations in Ensembl plants [68]. To identify the MYC TFs in wheat, rice, and *B. distachyon*, 7 known MYC proteins, including wheat TaMYC1, barley HvMyc1, rice OsMYC2, *Arabidopsis* MYC2, MYC3, MYC4, and AMS proteins sequences, were downloaded from Ensembl plants [68] and were used to build a hidden Markov model (HMM), then searched against the genome protein sequences of wheat, rice, *B. distachyon,* respectively, with a threshold of E< 1e-5 and the length of amino acid >200 aa (amino acid). Then, the HMM profile of the bHLH-MYC_N domain (PF14215) was downloaded from the Pfam database [69] to search against the protein sequences of wheat, rice, and *B. distachyon* with a threshold of E < 1e-5. After manual correction to remove the redundancy and alternative splice, the NCBI-CDD database (NCBI Conserved Domains Database) and SMART database (Simple Modular Architecture Research Tool) were used to confirm the putative MYC proteins. The ExPASy webserver [70] was used to predict the theoretical isoelectric point and molecular weight of TaMYCs, OsMYCs, and BdMYCs. To further verify the existence of *MYC* genes in wheat, rice, and *B. distachyon*, we performed BLASTN [71] to search for ESTs using the CDS of *MYC* genes. The CELLO web server [33] was used to predict the subcellular localization of MYC proteins.

### 4.2. Multiple Sequence Alignment and Phylogenetic Analysis of MYCs

An unrooted neighbor-joining (NJ) tree was constructed by MEGA 7.0 software with 1000 bootstrap replications and Jones–Taylor–Thornton model based on full-length protein sequence alignments was done by using the ClustalX 2.0 method [72,73]. The multiple sequence alignment was performed using the ClustalX 2.0 and visualized by Jalview [74], and the phylogenetic tree was visualized by Evolview [75].

### 4.3. Analysis of Gene Structures and Conserved Motifs

The exon–intron structure of *MYC* genes was graphically displayed by the Gene Structure Display Server [76] by using the CDS and DNA sequences of wheat, rice, and *B. distachyon MYC* genes. The protein sequences of MYCs were used to predict the conserved motifs by using the MEME online program [77] with the following parameters: optimum width of motifs set from 5 to 200 amino acids and maximum number of motifs set at 8. The gene structure and motifs were visualized by Evolview [75].

### 4.4. Analysis of Cis-Elements, Gene Duplication, and Synteny

The 2-kb upstream genomic DNA sequences of *MYC* genes were submitted to the PlantCARE online tool to identify the *cis*-elements [44]. Gene duplication and systeny analysis of MYCs in different species were done by using the MCScanX software [43] and visualized by Dual Systeny Plotter software by CJ-Chen [78].

### 4.5. Expression Profile Analysis

The cultivar of *T. aestivum* ‘Chinese Spring’, rice cultivar ‘Dongjin’, and *B. distachyon* Bd-21 were planted in an artificial climate chamber at 26/22 °C (day/night) with a photoperiod of 16/8 h (day/night). For tissues analysis, roots, stems, leaves, and inflorescences were collected at the heading stage. For different abiotic stresses, 2-week-old seedling plants were subjected to H_2_O (CK), heat (42 °C), cold (4 °C), drought (20% PEG6000), salt (200 mM NaCl), ABA (100 μM), and GA (100 μM) for 2 h, then whole plants were collected for RNA isolation. Total RNA was extracted using RNAiso Reagent (TaKaRa, Dalian, China) according to the manufacturer’s protocol and treated with DNaseI. The Transcriptor First Strand cDNA Synthesis Kit (Roche) was used to synthesize cDNA based on the manual. The QuantStudio^TM^ Real-Time PCR Software (ThermoFisher Scientific) was used to carry out qRT-PCR, data acquisition, and analysis. The volume of each reaction was 15 μL containing 7.5 μL of SYBR Premix Ex Taq (TaKaRa, Dalian, China), 0.75 μL (10 pmol μL^−1^) each of forward and reverse primers, 0.5 μL of cDNA (5.0 ng μL^−1^), and 5.5 μL of ddH_2_O. The reference genes (*Taactin*, *Osactin*, *Bdactin*) [79,80,81] were used to normalize the expression of the *TaMYC*, *OsMYC*, and *BdMYC* genes and the 2^(−ΔΔCt)^ analysis method was used to determine the relative expression level [82]. The following program was used for qRT-PCR: 50 °C for 2 min, 95 °C for 10 min, followed by 40 cycles of 95 °C for 15 s, 60 °C for 1 min in the PCR stage and 95 °C for 1 min, 95 °C for 15 s in the melt curve stage. Primers for 6 sets of *TaMYC* genes (*TaMYC3A/B/D*, *TaMYC4A/B/D*, *TaMYC1/5B/5D*, *TaMYC8A/B/D*, *TaMYC9A/B/D,* and *TaMYC10A/B/D*) were universal in each set, because of the highly conserved sequences in A, B, and D sub-genomes, thus the detected expression is the combination of three copies of homologous genes. The primers are listed in Table S6.

## 5. Conclusions

In this study, 26 TaMYC, 7 OsMYC, and 7 BdMYCTFs were identified and divided into five groups. The MYC proteins contained a JAZ interaction domain (JID) and a putative transcriptional activation domain (TAD) in the bHLH_MYC_N domain of the N-terminal region, the bHLH domain was found in the C-terminal region, some MYCs followed by a bHLH-ZIP domain. The expression profiles and identified three kinds of *cis*-elements indicate that MYC TFs function in plant development and in response to environmental stresses. Taken together, our results provide a solid foundation for further structural and functional investigations on MYCs.

## Supplementary Materials

The following data are available online at https://www.mdpi.com/2223-7747/8/8/274/s1, Figure S1: The JAZ interaction domain (JID), putative transcriptional activation domain (TAD) were identified in the MYC proteins; Figure S2: The bHLH domain and leucine zipper domain were identified in the MYC proteins; Table S1: The detailed information of *TaMYCs*, *OsMYCs*, *BdMYCs*; Table S2: The consensus ratio of the conserved amino acid residues in the bHLH_MYC_N domains, Table S3: The consensus ratio of the conserved amino acid residues in the bHLH domains of *TaMYCs*, *OsMYCs*, and *BdMYCs*, Table S4: Wheat, rice, and *B. distachyon MYC* gene duplication events, Table S5: The homologous genes between plant *MYC* genes, Table S6: Primer used in this study.

## References

[1] Nuno P., Liam D. (2010). Origin and diversification of basic-helix-loop-helix proteins in plants. Mol. Biol. Evol..

[2] Kazan K., Manners J.M. (2013). MYC2: The Master in Action. Mol. Plant.

[3] Ledent V., Vervoort M. (2001). The basic helix-loop-helix protein family: Comparative genomics and phylogenetic analysis. Genome Res..

[4] Xu Y.H., Liao Y.C., Lv F.F., Zhang Z., Sun P.W., Gao Z.H., Hu K.P., Sui C., Jin Y., Wei J.H. (2017). Transcription Factor AsMYC2 Controls the Jasmonate-responsive Expression of ASS1 Regulating Sesquiterpene Biosynthesis in Aquilaria sinensis (Lour.) Gilg. Plant Cell Physiol..

[5] Oikawa T., Maeda H., Oguchi T., Yamaguchi T., Tanabe N., Ebana K., Yano M., Ebitani T., Izawa T. (2015). The Birth of a Black Rice Gene and Its Local Spread by Introgression. Plant Cell.

[6] Tiancong Q., Jiaojiao W., Huang H., Bei L., Hua G., Yule L., Susheng S., Daoxin X. (2015). Regulation of Jasmonate-Induced Leaf Senescence by Antagonism between bHLH Subgroup IIIe and IIId Factors in Arabidopsis. Plant Cell.

[7] Gasperini D., Chételat A., Acosta I.F., Goossens J., Pauwels L., Goossens A., Dreos R., Alfonso E., Farmer E.E. (2015). Multilayered Organization of Jasmonate Signalling in the Regulation of Root Growth. PLoS Genet..

[8] Tiancong Q., Huang H., Susheng S., Daoxin X. (2015). Regulation of Jasmonate-Mediated Stamen Development and Seed Production by a bHLH-MYB Complex in Arabidopsis. Plant Cell.

[9] Gao C., Qi S., Liu K., Li D., Jin C., Li Z., Huang G., Hai J., Zhang M., Chen M. (2016). MYC2, MYC3, and MYC4 function redundantly in seed storage protein accumulation in Arabidopsis. Plant Physiol. Biochem..

[10] Zhu X., Chen J., Xie Z., Gao J., Ren G., Gao S., Zhou X., Kuai B. (2015). Jasmonic acid promotes degreening via MYC2/3/4- and ANAC019/055/072-mediated regulation of major chlorophyll catabolic genes. Plant J..

[11] Rajani S., Sundaresan V. (2001). The Arabidopsis myc/bHLH gene ALCATRAZ enables cell separation in fruit dehiscence. Curr. Biol..

[12] Heisler M., Atkinson A., Bylstra Y., Walsh R., Smyth D. (2001). SPATULA, a gene that controls development of carpel margin tissues in Arabidopsis, encodes a bHLH protein. Development.

[13] Anna-Marie S., Sandra K.B., Unte U.S., Peter H., Koen D., Heinz S. (2010). The Arabidopsis Aborted MicrosporeS (AMS) gene encodes a MYC class transcription factor. Plant J. Cell Mol. Biol..

[14] Na L., Da-Sheng Z., Hai-Sheng L., Chang-Song Y., Xiao-Xing L., Wan-Qi L., Zheng Y., Ben X., Huang-Wei C., Jia W. (2006). The rice tapetum degeneration retardation gene is required for tapetum degradation and anther development. Plant Cell.

[15] Cai Q., Yuan Z., Chen M., Yin C., Luo Z., Zhao X., Liang W., Hu J., Zhang D. (2014). Jasmonic acid regulates spikelet development in rice. Nat. Commun..

[16] Uji Y., Taniguchi S., Tamaoki D., Shishido H., Akimitsu K., Gomi K. (2016). Overexpression of OsMYC2 Results in the Up-Regulation of Early JA-Rresponsive Genes and Bacterial Blight Resistance in Rice. Plant Cell Physiol..

[17] Niu Y., Figueroa P., Browse J. (2011). Characterization of JAZ-interacting bHLH transcription factors that regulate jasmonate responses in Arabidopsis. J. Exp. Bot..

[18] Fabian S., Patricia F.C., Mark Z., Monica D.D., Sandra F., Gaétan G., Lewsey M.G., Ecker J.R., Roberto S., Philippe R. (2013). Arabidopsis basic helix-loop-helix transcription factors MYC2, MYC3, and MYC4 regulate glucosinolate biosynthesis, insect performance, and feeding behavior. Plant Cell.

[19] Zong Y., Xi X., Li S., Chen W., Zhang B., Liu D., Liu B., Wang D., Zhang H. (2017). Allelic Variation and Transcriptional Isoforms of WheatTaMYC1Gene Regulating Anthocyanin Synthesis in Pericarp. Front. Plant Sci..

[20] Shoeva O.Y., Mock H.P., Kukoeva T.V., Börner A., Khlestkina E.K. (2016). Regulation of the Flavonoid Biosynthesis Pathway Genes in Purple and Black Grains of Hordeum vulgare. PLoS ONE.

[21] Todd A.T., Enwu L., Polvi S.L., Pammett R.T., Page J.E. (2010). A functional genomics screen identifies diverse transcription factors that regulate alkaloid biosynthesis in Nicotiana benthamiana. Plant J..

[22] Yang N., Zhou W., Su J., Wang X., Li L., Wang L., Cao X., Wang Z. (2017). Overexpression of SmMYC2 Increases the Production of Phenolic Acids in Salvia miltiorrhiza. Front. Plant Sci..

[23] Lenka S.K., Nims N.E., Vongpaseuth K., Boshar R.A., Roberts S.C., Walker E.L. (2015). Jasmonate-responsive expression of paclitaxel biosynthesis genes in Taxus cuspidata cultured cells is negatively regulated by the bHLH transcription factors TcJAMYC1, TcJAMYC2, and TcJAMYC4. Front. Plant Sci..

[24] Abe H., Yamaguchi-Shinozaki K., Urao T., Iwasaki T., Hosokawa D., Shinozaki K. (1997). Role of arabidopsis MYC and MYB homologs in drought- and abscisic acid-regulated gene expression. Plant Cell.

[25] Shinozaki K., Yamaguchi-Shinozaki K. (2007). Gene networks involved in drought stress response and tolerance. J. Exp.Bot..

[26] Ohta M., Sato A., Na R. (2018). MYC-type transcription factors, MYC67 and MYC70, interact with ICE1 and negatively regulate cold tolerance in Arabidopsis. Sci. Rep..

[27] Lorenzo O., Chico JMSanchez-Serrano J.J., Solano R. (2004). Jasmonate-insensitive1 encodes a MYC transcription factor essential to discriminate between different jasmonate-regulated defense responses in Arabidopsis. Plant Cell.

[28] Chini A., Fonseca S., Fernández G., Adie B., Chico J.M., Lorenzo O., García-Casado G., López-Vidriero I., Lozano F.M., Ponce M.R. (2007). The JAZ family of repressors is the missing link in jasmonate signalling. Nature.

[29] Figueroa P., Browse J., Figueroa P., Browse J. (2015). Male sterility in Arabidopsis induced by overexpression of a MYC5-SRDX chimeric repressor. Plant J..

[30] Fernandez-Calvo P., Chini A., Fernandez-Barbero G., Chico J.M., Gimenez-Ibanez S., Geerinck J., Eeckhout D., Schweizer F., Godoy M., Franco-Zorrilla J.M. (2011). The Arabidopsis bHLH transcription factors MYC3 and MYC4 are targets of JAZ repressors and act additively with MYC2 in the activation of jasmonate responses. Plant Cell.

[31] Ogawa S., Kawahara-Miki R., Miyamoto K., Yamane H., Nojiri H., Tsujii Y., Okada K. (2017). OsMYC2 mediates numerous defence-related transcriptional changes via jasmonic acid signalling in rice. Biochem. Biophys. Res. Commun..

[32] Wusirika R., Jorge D., Yong-Jin P., Carlos B., John E., Phillip S.M., Bennetzen J.L. (2002). Different types and rates of genome evolution detected by comparative sequence analysis of orthologous segments from four cereal genomes. Genetics.

[33] Yu C.S., Chen Y.C., Hwang J.K. (2010). Prediction of protein subcellular localization. Proteins-Struct. Funct. Bioinform..

[34] Simionato E., Ledent V., Richards G., Thomas-Chollier M., Kerner P., Coornaert D., Degnan B.M., Vervoort M. (2007). Origin and diversification of the basic helix-loop-helix gene family in metazoans: Insights from comparative genomics. BMC Evol. Biol..

[35] Ledent V., Paquet O., Vervoort M. (2002). Phylogenetic analysis of the human basic helix-loop-helix proteins. Genome Biol..

[36] Zhang D., Li G., Wang Y. (2017). A genome-wide identification and analysis of basic helix-loop-helix transcription factors in cattle. Gene.

[37] Liu B., Song S., Huang H., Wang J., Qi T., Xie D. (2017). MYC5 is Involved in Jasmonate-Regulated Plant Growth, Leaf Senescence and Defense Responses. Plant Cell Physiol..

[38] Urao T., Yamaguchishinozaki K., Mitsukawa N., Shibata D., Shinozaki K. (1996). Molecular cloning and characterization of a gene that encodes a MYC-related protein in Arabidopsis. Plant Mol. Biol..

[39] Benson A., GenBank D. (2004). GenBank: update. Nucleic Acids Res..

[40] Bouchard C., Staller P., Eilers M. (1998). Control of Cell Proliferation by Myc. Trends Cell Biol..

[41] Xiaoxing L., Xuepeng D., Haixiong J., Yujin S., Yuanping T., Zheng Y., Jingkang G., Wanqi L., Liang C., Jingyuan Y. (2006). Genome-wide analysis of basic/helix-loop-helix transcription factor family in rice and Arabidopsis. Plant Physiol..

[42] Blackwood E., Eisenman R. (1991). Max: A helix-loop-helix zipper protein that forms a sequence-specific DNA-binding complex with Myc. Science.

[43] Niu X., Guan Y., Chen S., Li H. (2017). Genome-wide analysis of basic helix-loop-helix (bHLH) transcription factors in Brachypodium distachyon. BMC Genom..

[44] Yupeng W., Haibao T., Debarry J.D., Xu T., Jingping L., Xiyin W., Tae-Ho L., Huizhe J., Barry M., Hui G. (2012). MCScanX: A toolkit for detection and evolutionary analysis of gene synteny and collinearity. Nucleic Acids Res..

[45] Magali L., Patrice D., Gert T., Kathleen M., Yves M., Yves V.D.P., Pierre R., Stephane R. (2002). PlantCARE, a database of plant cis-acting regulatory elements and a portal to tools for in silico analysis of promoter sequences. Nucleic Acids Res..

[46] Giuliano G., Pichersky E., Malik V.S., Timko M.P., Scolnik P.A., Cashmore A.R. (1988). An evolutionarily conserved protein binding sequence upstream of a plant light-regulated gene. Proc. Natl. Acad. Sci. USA.

[47] Haidar M.A., Henning D., Busch H. (1991). Sp1 is essential and its position is important for p120 gene transcription: A 35 bp juxtaposed positive regulatory element enhances transcription 2.5 fold. Nucleic Acids Res..

[48] Carlini L.E., Ketudat M., Parsons R.L., Prabhakar S., Schmidt R.J., Guiltinan M.J. (1999). The maize EmBP-1 orthologue differentially regulates Opaque2-dependent gene expression in yeast and cultured maize endosperm cells. Plant Mol. Biol..

[49] Zhang L.F., Li W.F., Han S.Y., Yang W.H., Qi L.W. (2013). cDNA cloning, genomic organization and expression analysis during somatic embryogenesis of the translationally controlled tumor protein (TCTP) gene from Japanese larch (Larix leptolepis). Gene.

[50] He Y., Gan S. (2001). Identical promoter elements are involved in regulation of the OPR1 gene by senescence and jasmonic acid in Arabidopsis. Plant Mol. Biol..

[51] Zheng Z., Xiaoming Y., Yaping F., Longfei Z., Hantian W., Xinchun L. (2017). Overexpression of PvPin1, a Bamboo Homolog of PIN1-Type Parvulin 1, Delays Flowering Time in Transgenic Arabidopsis and Rice. Front. Plant Sci..

[52] Ezcurra I., Ellerström M., Wycliffe P., Stålberg K., Rask L. (1999). Interaction between composite elements in the napA promoter: Both the B-box ABA-responsive complex and the RY/G complex are necessary for seed-specific expression. Plant Mol. Biol..

[53] Sutoh K., Yamauchi D. (2010). Two cis-acting elements necessary and sufficient for gibberellin-upregulated proteinase expression in rice seeds. Plant J..

[54] Liu Z.-B. (1994). Soybean GH3 promoter contains multiple auxin-inducible elements. Plant Cell.

[55] Ballas N., Wong L.-M., Theologis A. (1993). Identification of the Auxin-responsive Element, AuxRE, in the Primary indoleacetic Acid-inducible Gene, PS-IAA4/5, of Pea (Pisum sativum). J. Mol. Biol..

[56] Goldsbrough A.P., Albrecht H., Stratford R. (2010). Salicylic acid-inducible binding of a tobacco nuclear protein to a 10 bp sequence which is highly conserved amongst stress-inducible genes. Plant J. Cell Mol. Biol..

[57] Zhang Z.Y., Zhao J., Hu Y., Zhang T.Z. (2015). Isolation of GhMYB9 gene promoter and characterization of its activity in transgenic cotton. Biol. Plant..

[58] Dunn M.A., White A.J., Vural S., Hughes M.A. (1998). Identification of promoter elements in a low-temperature-responsive gene (blt4.9) from barley (Hordeum vulgare L.). Plant Mol. Biol..

[59] Geffers R., Sell S., Cerff R., Hehl R. (2001). The TATA box and a Myb binding site are essential for anaerobic expression of a maize GapC4 minimal promoter in tobacco. Biochim. Biophys. Acta.

[60] Estes K.S., Anderson D.G., Stoler D. (1995). Anoxic induction of a sarcoma virus-related VL30 retrotransposon is mediated by a cis-acting element which binds hypoxia-inducible factor 1 and an anoxia-inducible factor. J. Virol..

[61] Feller A., Machemer K., Braun E.L., Grotewold E. (2011). Evolutionary and comparative analysis of MYB and bHLH plant transcription factors. Plant J. Cell Mol. Biol..

[62] Amoutzias G.D., Robertson D.L., Peer Y.V., Oliver S.G. (2008). Choose your partners: Dimerization in eukaryotic transcription factors. Trends Biochem. Sci..

[63] Chen R., Jiang H., Li L., Zhai Q., Qi L., Zhou W., Liu X., Li H., Zheng W., Sun J. (2012). The Arabidopsis Mediator Subunit MED25 Differentially Regulates Jasmonate and Abscisic Acid Signaling through Interacting with the MYC2 and ABI5 Transcription Factors. Plant Cell..

[64] Baxevanis A.D., Vinson C.R. (1993). Interactions of coiled coils in transcription factors: Where is the specificity?. Curr. Opin. Genet. Dev..

[65] Wei K., Chen H. (2018). Comparative functional genomics analysis of bHLH gene family in rice, maize and wheat. BMC Plant Biol..

[66] Bruno D., Ping X.G., Sprague S.J., Kirkegaard J.A., Ross J.J., Reid J.B., Fitt G.P., Nasser S., Schenk P.M., Manners J.M. (2007). MYC2 differentially modulates diverse jasmonate-dependent functions in Arabidopsis. Plant Cell.

[67] Hongtao Z., Sabah H., Grégory M., Yanxia Z., Guillaume C., Martial P., Pascal G., Johan M. (2011). The basic helix-loop-helix transcription factor CrMYC2 controls the jasmonate-responsive expression of the ORCA genes that regulate alkaloid biosynthesis in Catharanthus roseus. Plant J..

[68] Dan M.B., Staines D.M., Perry E., Kersey P.J. (2017). Ensembl Plants: Integrating Tools for Visualizing, Mining, and Analyzing Plant Genomic Data. Methods Mol. Biol..

[69] Finn R.D., John T., Jaina M., Coggill P.C., Stephen John S., Hans-Rudolf H., Goran C., Kristoffer F., Eddy S.R., Sonnhammer E.L.L. (2008). The Pfam protein families database. Nucleic Acids Res..

[70] Panu A., Manohar J., Konstantin A., Delphine B., Gabor C., Edouard D.C., Séverine D., Volker F., Arnaud F., Elisabeth G. (2012). ExPASy: SIB bioinformatics resource portal. Nucleic Acids Res..

[71] Johnson M., Zaretskaya I., Raytselis Y., Merezhuk Y., McGinnis S., Madden T.L. (2008). NCBI BLAST: A better web interface. Nucleic Acids Res..

[72] Kumar S., Stecher G., Tamura K. (2016). MEGA7: Molecular Evolutionary Genetics Analysis Version 7.0 for Bigger Datasets. Mol. Biol. Evol..

[73] Larkin M., Blackshields G., Brown N., Chenna R., Mcgettigan P., Mcwilliam H., Valentin F., Wallace I., Wilm A., Lopez R. (2007). Clustal W and clustal X version 2.0. Bioinformatics.

[74] Waterhouse A., Procter J., Martin D.A., Barton G.J. (2005). Jalview: Visualization and Analysis of Molecular Sequences, Alignments, and Structures. BMC Bioinform..

[75] He Z., Zhang H., Gao S., Lercher M.J., Chen W.H., Hu S. (2016). Evolview v2: An online visualization and management tool for customized and annotated phylogenetic trees. Nucleic Acids Res..

[76] Hu B., Jin J., Guo A.Y., Zhang H., Luo J., Gao G. (2014). GSDS 2.0: An upgraded gene feature visualization server. Bioinformatics.

[77] Bailey T.L., Nadya W., Chris M., Li W.W. (2006). MEME: Discovering and analyzing DNA and protein sequence motifs. Nucleic Acids Res..

[78] Liu C., Xie T., Chen C., Luan A., Long J., Li C., Ding Y., He Y. (2017). Genome-wide organization and expression profiling of the R2R3-MYB transcription factor family in pineapple (Ananas comosus). BMC Genom..

[79] Hong S.Y., Seo P.J., Yang M.-S., Xiang F., Park C.-M. (2008). Exploring valid reference genes for gene expression studies inBrachypodium distachyonby real-time PCR. BMC Plant Biol..

[80] Ramesh S.A., Kamran M., Sullivan W., Chirkova L., Okamoto M., Degryse F., McLauchlin M., Gilliham M., Tyerman S.D. (2018). Aluminium-Activated Malate Transporters Can Facilitate GABA Transport. Plant Cell.

[81] Kim B.R., Nam H.-Y., Kim S.-U., Kim S.-I., Chang Y.-J. (2003). Normalization of reverse transcription quantitative-PCR with housekeeping genes in rice. Biotechnol. Lett..

[82] Livak K.J., Schmittgen T.D. (2001). Analysis of Relative Gene Expression Data Using Real-Time Quantitative PCR and the 2−ΔΔ C T Method. Methods.

